# Caffeine and taurine: a systematic review and network meta-analysis of their individual and combined effects on physical capacity, cognitive function, and physiological markers

**DOI:** 10.1080/15502783.2025.2566371

**Published:** 2025-10-01

**Authors:** Hengzhi Deng, Li Wang, Ping Liu, Mohamed Nashrudin Bin Naharudin, Xiaohan Fan

**Affiliations:** a University Malaya, Faculty of Sports and Exercise Science, Kuala Lumpur, Malaysia; b Shanghai Ocean University, Department of Physical Education and Sport, Shanghai, China

**Keywords:** Caffeine, taurine, co-supplementation, physical capacity, metabolic response

## Abstract

**Background:**

Caffeine (CAF) and taurine (TAU) have each demonstrated ergogenic effects across physical and cognitive domains. Often co-formulated in commercial energy drinks, they are widely regarded as the two principal bioactive compounds. However, findings regarding their combined efficacy remain inconclusive. This systematic review and Bayesian network meta-analysis aimed to quantify the individual and combined effects of CAF and TAU on physical capacity, cognitive function, and physiological responses, with a focus on identifying potential synergistic or antagonistic interactions.

**Methods:**

Cochrane Library, PubMed, SciELO, SportsDiscus-EBSCO and Web of Science were searched through 25 July 2025. The pooled effect of each outcome was summarized using SMD (Hedge’s g) by Bayesian arm-based multilevel network meta-analysis, and SUCRA ranking was applied to estimate the relative treatment effect.

**Results:**

Twelve studies were included (8 on physical capacity, 7 on blood lactate (B[la]), and 6 each on cognitive function, heart rate (HR), and rating of perceived exertion (RPE)). Posterior estimates indicated that CAF+TAU was associated with a credible positive effect on anaerobic capacity (g = 0.46, 95% CrI [0.19, 0.71]) and reaction time (g = 0.75, 95% CrI [0.29, 1.18]) compared to CAF or TAU alone. CAF showed the greatest posterior reduction in RPE (g = -0.64, 95% CrI [−1.20, −0.10]), while its posterior mean estimate suggested a potential increase in B[la] (g = 0.24, 95% CrI [−0.48, 0.96]). In contrast, TAU showed a possible tendency toward reducing B[la] (g = -0.30, 95% CrI [−1.01, 0.42]). No credible differences in HR were observed across conditions. Effects on aerobic performance and physiological measures were variable and appeared to be context-dependent. SUCRA rankings consistently favored CAF+TAU across most outcome domains.

**Conclusions:**

CAF+TAU co-supplementation provides a balanced ergogenic effect, combining the central stimulation of CAF with the neuromodulatory and metabolic support of TAU, particularly beneficial for high-intensity, reaction-based tasks. Its effects on endurance and physiological indices vary by condition, highlighting the need for personalized application.

## 1. Introduction

Caffeine (CAF) (1,3,7-trimethylxanthine) and taurine (TAU) (2-aminoethanesulfonic acid) are two of the most widely consumed ergogenic aids in both sports nutrition and everyday energy products [1,2]. Caffeine, a well-studied central nervous system stimulant, has been consistently reported to enhance exercise performance by reducing perceived exertion, improving endurance, and increasing power output [3,4]. Its mechanisms of action include adenosine receptor antagonism, increased catecholamine release, and enhanced calcium handling in muscle cells [5]. On the other hand, taurine, a conditionally essential sulfur-containing amino acid, plays a critical role in cellular hydration, calcium signaling, antioxidation, and the maintenance of proper muscle function [6,7]. These properties make taurine a key component in supporting exercise performance and recovery, particularly under conditions of physical stress or high-intensity exercise [8].

In recent years, the simultaneous supplementation of performance-enhancing aids has emerged as a popular strategy in sports nutrition [9,10]. Athletes and active individuals often combine multiple compounds to target diverse physiological pathways, aiming to maximize performance gains and accelerate recovery. The combination of caffeine and taurine is particularly intriguing due to their distinct yet potentially complementary mechanisms of action. While caffeine primarily enhances alertness and reduces fatigue through central nervous system stimulation, taurine may mitigate oxidative stress, improve muscle contractility, and support cellular homeostasis [7,11]. This synergy could theoretically lead to greater performance benefits than either compound alone. However, the interaction between these substances may also result in unforeseen or counterproductive outcomes, such as increased heart rate (HR), altered energy metabolism, or adverse cardiovascular effects [12,13]. Therefore, a nuanced understanding of their combined effects is essential to optimize their use and ensure safety.

The growing popularity of energy drinks containing caffeine and taurine as a means to enhance exercise performance further underscores the need for rigorous scientific investigation [14]. These beverages are marketed not only to athletes but also to the general population, often claiming to boost energy and improve physical performance [1,2]. While caffeine and taurine are frequently cited as the primary active ingredients responsible for these benefits, the overall composition of energy drinks is complex, typically including additional compounds such as vitamins, minerals, amino acids, and other stimulants [15,16]. This complexity makes it challenging to isolate the specific effects of caffeine and taurine and determine whether their combination truly provides synergistic or additive benefits.

Despite the widespread use of caffeine and taurine, both individually and in combination, the scientific literature on their combined effects remains limited and inconsistent. While some studies suggest potential synergies in enhancing exercise performance and cognitive function, others report no significant additional benefits compared to caffeine or taurine alone [17–20]. Furthermore, the physiological impacts of co-supplementation, such as effects on blood pressure, HR, and metabolic markers, are not well understood. This gap in knowledge highlights the need for a comprehensive synthesis of existing evidence to clarify the efficacy and safety of caffeine and taurine co-supplementation.

Therefore, this systematic review and network meta-analysis aim to evaluate the effects of co-supplementation of caffeine and taurine on exercise performance, cognitive function, and key physiological indicators such as HR and blood lactate (B[la]). By comparing the combined effects of caffeine and taurine to their isolated intake, this study seeks to provide evidence-based insights into their potential synergies or antagonisms. The findings will not only contribute to the optimization of sports nutrition strategies but also inform the formulation of energy products and provide practical recommendations for their safe and effective use.

## 2. Methods

For this systematic review, we followed Preferred Reporting Items for Systematic Reviews and Meta-analysis (PRISMA) guidelines [21].

### 2.1. Inclusion/exclusion criteria

Studies were selected according to the following inclusion criteria: Included only studies in humans; Published as a full-text in English and in a peer-reviewed journal; Experimental design with at least one session combining the ingestion of CAF and TAU; The outcomes examined were those related to exercise performance and physiological responses, such as HR and B[la]; Finally, the design of the included studies had to be at least a single-blind randomized controlled trial.

The following studies were excluded: animal or in vitro cell-based studies, studies published only as conference abstracts, dissertations, or other forms of gray literature and studies conducted in diseased or injured subjects. The primary reason for excluding studies was the co-ingestion of additional supplements alongside CAF and TAU, such as amino acids, glucose, or carbohydrates.

### 2.2. Literature search

Records were retrieved by searching for studies using the databases Cochrane Library, PubMed, SciELO, SportsDiscus-EBSCO and Web of Science. The terms used for the literature search were: concept 1 (caffeine) AND concept 2 (taurine) AND concept 3 (ergogenic* OR performance* OR combination*). The searches were conducted on 25 March 2025, and updated on 25 July 2025. The search was conducted without any publication year restriction, and no filters were used. All titles and abstracts from the search were downloaded to a Microsoft Excel spreadsheet and manual cross-referencing was performed to identify duplicates.

### 2.3. Study selection

A two-stage search strategy was carried out after duplicates were removed. Firstly, the researchers read the title and abstract; studies with a title and/or abstract that did not meet the inclusion criteria were excluded. Secondly, the remaining articles were fully read, those that did not meet the inclusion criteria were removed.

### 2.4. Data extraction

Two authors (D.H.Z and F.X.H) extracted from the included studies the following data: study source (authors and year of publication), experimental design (type of study), sample characteristics (number of participants, gender, different supplementation groups, age, and training status), supplementation characteristics (type, dose and timing) and type of physical capacity, cognitive function and physiological responses (test performed for evaluation, discipline, time or distance, bouts, rest type and time).

### 2.5. Risk of bias and quality of methods assessment

The risk of bias was assessed using the Cochrane Collaboration’s Risk of Bias tool 2 (Rob2) [22], which evaluates random sequence generation, random allocation concealment, blinding of outcome assessment, incomplete outcome data, and selective outcome reporting. Disagreements were resolved through discussion whenever possible. If consensus could not be reached, a third reviewer (M.N.N) acted as an arbitrator. Additionally, the methodological quality of the selected studies was analyzed using the QualSyst tool [23]. The QualSyst tool consists of 14 items assessing various methodological aspects of a given study, from “Question/objective sufficiently described?” until “Conclusions supported by the results?” This tool was chosen due to the broad assessment of methodological aspects of a study. Two researchers independently attributed Yes ( = 2), Partial ( = 1), No ( = 0) or not applicable (N/A) for each item. A summary score was calculated using the following calculation:

summary score = total sum/possible sum

where the total sum is the number of “yes” multiplied by 2 plus the number of “partial” multiplied by 1. The total possible sum is 28 minus the number of “N/A” multiplied by 2. Studies with summary scores of > 0.75 were considered as “strong,” between 0.55 − 0.75 as “moderate” and < 0.55 as “weak.” Differences of opinion between the two researchers were debated and a common score was attributed; when the difference was unresolved, the same third reviewer served as the arbitrator and assigned the final score.

### 2.6. Statistical analysis

To summarize our analytical strategy, we first calculated standardized effect sizes (Hedge’s g) for each outcome. These were then synthesized using a Bayesian arm-based network meta-analysis to compare CAF, TAU, and CAF+TAU against placebo and each other. We additionally examined treatment ranking, heterogeneity, and potential bias to ensure both statistical rigor and interpretability.

#### 2.6.1. Data synthesis and effect measures

Considering the impact of the number of studies, the data extraction process for the meta-analysis focused on the results of physical capacity, rating of perceived exertion (RPE), cognitive ability, and physiological indicators (HR, B[la]). For studies with pretest and posttest data, the standardized mean difference (SMD) and standard error (SE) were calculated using the difference values provided in the article. For differences not provided or other required data, we contacted the corresponding author or extracted them from the images using the WebPlotDigitizer program.

In this study, we explored the optimal regimen by comparing CAF, TAU, and CAF+TAU with placebo treatment. Among the 12 included studies, except for the Çalışkan et al. study which was a parallel experimental design [13], the other studies were crossover experimental designs. The mean difference (MD) and the standard deviation of the change in means (SD_pooled_) were calculated according to the recommendations of the Cochrane handbook for the evaluation of intervention systems (Version 6.5, 2024), using the following formula [24]. The first step involved calculating the difference in means:

$$\mathrm{MD}=M_{\mathrm{EXP}}-M_{\mathrm{PLA}}$$

Where the M_EXP_ represents the mean value of the experimental groups (CAF group, TAU group or CAF+TAU group), M_PLA_ represents the mean value of the placebo group.

Then the SD_pooled_ for crossover experiments studies was calculated as follows [25]:

$$SD_{\mathrm{pooled}}=\sqrt{\frac{S{D^{2}}_{\mathrm{EXP}}+S{D^{2}}_{\mathrm{PLA}}}{2}}$$

Where SD_EXP_ is the standard deviation from experimental groups, and SD_PLA_ is the standard deviation from placebo group. This formula accounts for the fact that in crossover designs, the same participants serve as their own controls, reducing variability between conditions.

On the other hand, the SD_pooled_ for parallel experiments studies was calculated as follows [25]:

$$SD_{\mathrm{pooled}}=\sqrt{\frac{\left(n_{\mathrm{EXP}}-1\right)\times SD_{EXP}^{2}+\left(n_{\mathrm{PLA}}-1\right)\times SD_{PLA}^{2}}{n_{\mathrm{EXP}}+n_{\mathrm{PLA}}-2}}$$

Where n_EXP_ is the sample sizes from experimental group, and n_PLA_ is the sample sizes from placebo group. This formula weights the standard deviations by their respective sample sizes to provide a more accurate combined estimate.

Considering the relatively small sample sizes in most included studies, Hedge’s g (g) was used as the mean effect size point estimate in each analysis. Hedge’s g provides a bias-corrected version of Cohen’s d that is more accurate for small samples. For crossover experimental design, it was calculated using the following formula [25]:

$$\mathrm{Hedg}e^{\prime }sg=\;\frac{\left(\;[M_{\mathrm{EXP}}\right]-\;[M_{\mathrm{PLA}}])}{SD_{\mathrm{pooled}}}\times \left(1-\frac{3}{4\left(N-1\right)-1}\right)$$

Where *N* is the total sample sizes.

For the parallel experimental design, the g was calculated using the following formula [25]:

$$\mathrm{Hedg}e^{\prime }sg=\;\frac{\left(\;[M_{\mathrm{EXP}}\right]-\;[M_{\mathrm{PLA}}])}{SD_{\mathrm{pooled}}}\times \left(1-\frac{3}{4\left(n_{\mathrm{EXP}}+n_{\mathrm{PLA}}-2\right)-1}\right)$$

g was classified as *trivial* (0.2), *small* (0.2–0.5), *medium* (0.5–0.8), and *large* ( > 0.8) [26].

For the crossover experimental design, the SE of g was calculated using the following formula [25]:$\mathrm{SE}=\sqrt{\frac{1}{N}+\frac{g^{2}}{2N}}\times \sqrt{2\left(1-r\right)}$

Where *r* is the correlation coefficient between the experimental and placebo group measurements. Since this correlation was rarely reported in the included studies, it was generally assumed to be *r* = 0.50, as recommended by the Cochrane Handbook [27]. This correlation coefficient reflects the degree to which participants’ responses are consistent across treatment conditions. A moderate correlation (*r* = 0.50) is a reasonable assumption when individual-level correlations are unavailable.

For the parallel experimental design, the SE of g was calculated using the following formula [28]:

$$\mathrm{SE}=\sqrt{\frac{n_{\mathrm{EXP}}+n_{\mathrm{PLA}}}{n_{\mathrm{EXP}}\times n_{\mathrm{PLA}}}+\frac{g^{2}}{2\left(n_{\mathrm{EXP}}+n_{\mathrm{PLA}}\right)}}$$

#### 2.6.2. Network meta-analysis

All network meta-analytical approaches allow users to assess the effects of treatments against a range of comparisons. For main and moderation analyses, we used Bayesian arm-based multilevel network meta-analysis models [29]. This approach differs from traditional contrast-based network meta-analysis by modeling the absolute effects of each treatment arm rather than pairwise comparisons, which is particularly advantageous when all treatments share a common comparator (placebo in our case).

Specifically, Bayesian meta-analysis was performed in R using the brms package. We nested effects within groups to manage the dependencies between multiple effect sizes from the same participants [30]. The Bayesian approach provides probability distributions for effect estimates rather than point estimates, allowing for more nuanced interpretation of uncertainty.

Arm based meta-analyses instead describe the population-averaged absolute effect size for each treatment arm (i.e. each arm’s change score) [29]. Our effect sizes were computed based on differences between experimental groups (CAF, TAU, CAF+TAU) and the placebo group, and the result already represented the posterior estimated mean and its 95% credible interval (CrI) of the corresponding experimental groups. The 95% CrI represents the range within which the true effect is expected to lie with 95% probability. At the same time, the network geometry diagram we drew using the *netmeta* package no longer includes the placebo group, and its main function is to demonstrate the transitivity between different experimental groups.

Next, we directly extracted the effect size differences between CAF, TAU, and CAF+TAU relative to placebo from the Bayesian model using the *emmeans* package to help find the best solution [31]. These treatment contrasts were accompanied by 95% highest posterior density (HPD) intervals, representing the narrowest intervals of the posterior distribution that contain 95% of its total probability mass [31]. HPD intervals provide the most credible range of effect sizes given the observed data. Then, we ran the surface under the cumulative ranking curve test (SUCRA) using the *netmeta* package in R to estimate the relative ranking of treatments. This approach allowed us to assess the relative efficacy of each experimental condition while incorporating the uncertainty inherent in the treatment rankings. SUCRA values range from 0 to 1, where higher values indicate a greater likelihood that the treatment is the most effective [30].

In addition, we calculated the prediction interval (PI) for each experimental group under different indicators to evaluate the practical application value of the results and explicitly quantify the impact of heterogeneity [24,25]. Following recommendations from the PRISMA guidelines and methodological frameworks for random-effects meta-analyses, the PI incorporates both the uncertainty in the pooled effect estimate (parameter estimation error) and the between-study heterogeneity (τ^2^) [26,27]. Specifically, the width of the PI directly reflects the magnitude of heterogeneity: a wider PI indicates higher variability in true effects across studies, suggesting that future research may yield results substantially different from the current pooled estimate [27]. To calculate the PI, we simulated potential effects of new studies from the posterior distribution of the Bayesian hierarchical model, capturing the full impact of estimation uncertainty (expressed through credible intervals) and between-study variability (quantified by τ^2^) [24,32]. This approach ensures that the PI accounts for both sources of variability, providing a realistic range of effect sizes that might be observed in future clinical settings [28].

Finally, we calculated the minimal clinically important difference (MCID, 0.5 standard deviation of the change score) for the experimental groups relative to the placebo group under different indicators and compared the absolute effect size with the MCID to assess whether the experimental intervention was clinically meaningful [33]. An effect size exceeding the MCID suggests that the treatment difference is large enough to be noticeable and relevant to practitioners and participants.

#### 2.6.3. Moderator analysis

The Bayesian arm-based model facilitated the examination of hypothesized moderators, allowing us to conduct a moderator analysis on both motor and cognitive performance indicators [29]. Moderator analysis examines whether treatment effects vary systematically across different types of outcomes or study characteristics. According to Mitchell et al. [34] recommendations, exercise performance was categorized into aerobic and anaerobic capacity, while cognitive performance was simply stratified into reaction time and other cognitive measures. Due to the limited number of included studies, further subgroup analyses were not conducted. Additionally, no moderator analysis was applied to the results of HR, B[la], and RPE due to insufficient data diversity.

#### 2.6.4. Risk of publication bias and sensitivity analysis

We generated contour-enhanced funnel plots for each primary analysis outcome across different indicators and conducted multilevel Egger regression tests to assess potential publication bias [35–37]. Contour-enhanced funnel plots help visualize whether study effects are distributed symmetrically around the pooled estimate, with asymmetry potentially indicating publication bias. Additionally, we computed the S-value, which quantifies the degree of publication bias required to nullify the meta-analytic effect, thereby evaluating the robustness of the findings [38]. Higher S-values indicate that substantial publication bias would be needed to eliminate the observed treatment effects.

Since effect sizes were calculated from the difference between the experimental groups and the placebo group, the direct incoherence assessment between experimental groups and placebo group was not applicable. However, our primary aim was to find the most effective treatment, and to validate the results of the arm-based multilevel network meta-analysis and the rankings derived from SUCRA, we attempted to assess the consistency of comparisons between experimental groups using placebo group as a common benchmark. Network consistency refers to the agreement between direct evidence (from head-to-head comparisons) and indirect evidence (inferred through common comparators). Specifically, we employed two methods in the *netmeta* package: 1) Global design-by-treatment interaction test: To assess network-wide inconsistency across all comparisons by testing whether treatment effects vary significantly across different study designs. 2) Node-splitting method: To examine local inconsistency for each pair of experimental groups (e.g. CAF vs. TAU) by separating direct and indirect evidence (if available).

## 3. Results

### 3.1. Study selection

**Figure 1.**
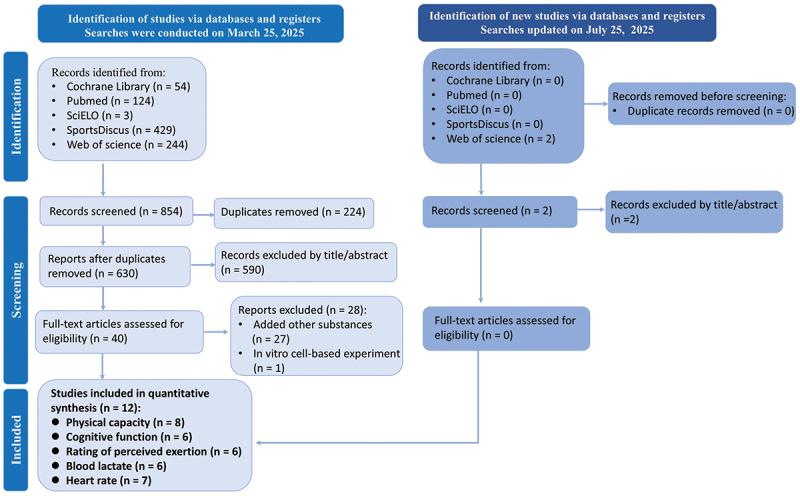
PRISMA flow diagram for included and excluded studies.*

The initial search yielded 856 publications: 854 from the primary database search, 2 from an updated search conducted three months later. After screening, 12 studies were deemed eligible to be included in the systematic review and meta-analysis: 8 for physical capacity, 6 for cognitive function, RPE and B[la], 7 for HR. The PRISMA flow diagram outlines the study selection process (Figure 1).

### 3.2. Characteristics of included studies

All included studies were randomized controlled trials. The total number of participants was 228, including 134 males, 76 females, and 18 participants whose gender was not reported. The sample size of each study ranged from 7 to 56 participants, and the age of all participants were over 18 years old. Based on the descriptions provided in the original studies, participants were categorized into two groups according to their physical activity status: (1) Untrained or unspecified individuals, including healthy untrained students [13,18,39], or university students whose training status was not clearly specified [12,40]; and (2) Trained individuals, including moderately trained university students [19,41–43], athletes [6], elite boxers [17], and soldiers undergoing structured physical training [20]. Additional details on participants and intervention protocols are provided in Tables 1 and 2.

**Table 1.** Results and characteristics of individual studies on physical capacity.

Study and design	Test protocol	Sample	Training status	Age (y); Weight (kg)	Dosage; Form; Timing	Washout period	Outcomes	Statistical significance (*p* < 0.05)
Aggarwal et al. [39] RSB Crossover	Surgical simulations on MIST-VR (MD and SD)	18 NS	Healthy untrained students	>18; N/A	C: 150 mg C+T: 150 mg +2 g PLA: vitamin C and calcium capsule (NS); Capsule; 1 h before	Min 48 h	MD, SD time (s): C: 37.5 ± 8.89, 41 ± 5.93 vs C+T: 34.25 ± 6.67, 40.25 ± 5.19 vs P: 40 ± 7.41, 42 ± 7.41 MD, SD scores: C: 3.32 ± 0.57, 4.43 ± 0.84 vs C+T: 3.08 ± 0.37, 4.4 ± 1.02 vs P: 3.25 ± 0.43, 4.24 ± 1.07 MD, SD error score: C: 64.5 ± 14.81, 5 ± 4.44 vs C+T: 69.5 ± 31.11, 4.75 ± 2.22 vs P: 66.5 ± 37.04, 4.75 ± 3.7	N/A
Jeffries et al. [19] RDB Crossover	10 × 6s cycling sprint, with 24s rest interval	11 males	Physically active university males (Min training 4 h/week)	21 ± 2; 80 ± 13	C+T: 80 mg +1000 mg P: 1080 mg maltodextrin; Capsule; 1 h before	1 week	Peak power (W): C+T: 1011 ± 87 vs P: 1006 ± 84 Rate of intra fatigue (%): C+T: 44 ± 19 vs P: 35 ± 13 Rate of inter fatigue (%): C+T: 14 ± 3 vs P: 16 ± 5.1 Baseline, sprint 5 and ending heart rate (bpm): C+T: 140 ± 16, 173 ± 10 and 174 ± 10 vs P: 127 ± 20, 170 ± 9 and 174 ± 10 Baseline, sprint 5 and ending B[la] (mmol/L): C+T: 1.1 ± 0.3, 6.7 ± 1.0 and 10.8 ± 1.4 vs P: 1.0 ± 0.2, 6.0 ± 0.7 and 10.2 ± 1.2 Baseline, sprint 5 and ending RPE: C+T: 10 ± 4, 16 ± 3 and 19 ± 1 vs P: 10 ± 3, 15 ± 2 and 19 ± 1	N/A
Kammerer et al. [20] RDB Crossover	Ramp running TTE, Handgrip strength, vertical jump	14 males	Healthy trained soldiers	20 ± 1; 68 ± 7.2	C: 80 mg T: 1000 mg C+T: 80 mg +1000 mg PLA: Similar flavor carbonated-based water; Beverage; 45 min before	48–72 h	TTE (min): C: 17 ± 1.03 vs T: 16.9 ± 1.4 vs C+T: 16.8 ± 1.2 vs P: 17 ± 1.2 Right hand strength (kg): C: 55.3 ± 6.7 vs T:55.4 ± 6.9 vs C+T: 57.5 ± 6.2 vs P: 56.8 ± 6.6 Left hand strength (kg): C: 53.5 ± 4.9 vs T: 52.6 ± 7 vs C+T: 54 ± 6.3 vs P: 53.1 ± 5.9 Vertical jump (cm): C: 41.7 ± 3.8 vs T: 42.5 ± 4.4 vs C+T : 41.5 ± 4.2 vs P: 41.1 ± 3.8 HRmax (bpm): C: 196.5 ± 4.5 vs T: 197.8 ± 6.6 vs C+T: 197.1 ± 6 vs P: 196 ± 6.8	No
Karayigit et al. [6] RDB Crossover	30-s Wingate test (WanT)	17 females	Team sport athletes (Min training 4 times/week)	23.4 ± 2.1; 59.5 ± 2.2	C: 6 mg/kg T: 1 g C+T: 6 mg/kg BM +1 g PLA: 300 mg maltodextrin; Capsule; 1 h before	Min 48 h	Peak power (W): C: 702.4 ± 95.38 vs T: 688.35 ± 91.69 vs C+T: 715.71 ± 99.08 vs P: 680.96 ± 86.51 Mean power (W): C: 498.29 ± 78.04 vs T: 493.36 ± 65.53 vs C+T: 504.05 ± 76.76 vs P: 495.29 ± 73.87 Fatigue index (%): C: 28.95 ± 6.56 vs T: 28.23 ± 5.77 vs C+T: 29.74 ± 5.77 vs P: 28.03 ± 6.04 B[la] (mmol/L): C: 8.0 ± 1.7 vs T: 7.5 ± 1.3 vs C+T: 7.8 ± 1.1 vs P: 7.7 ± 1.4 Heart rate (bpm): C: 179.5 ± 6.1 vs T: 179.0 ± 4.6 vs C+T: 180.2 ± 4.0 vs P: 179.8 ± 4.1 RPE: C: 16.5 ± 1.5 vs T: 17.0 ± 1.6 vs C+T: 16.6 ± 1.9 vs P: 16.8 ± 1.6	Yes only with C+T vs P in mean power and C+T vs P/T in peak power↑
Liu & Rong [41] RDB Crossover	Incremental cycling TTE in hypoxia, CMJ, 6 × 10 s repetitive sprints in hypoxia; Stroop test	16 males	University football players (training 3.63 ± 1.27 years)	23.7 ± 2.2; 75.0 ± 7.8	C: 5 mg/kg T: 50 mg/kg C+T: 5 mg/kg +50 mg/kg PLA: 5 mg/kg maltodextrin; Capsule; 1 h before	3 days	TTE (s): C: 618.56 ± 42.5 vs T: 584.19 ± 67.59 vs C+T: 613.69 ± 37.37 vs P: 566.94 ±v 54.35 CMJ (cm): C: 44.11 ± 4.72 vs T: 43.42 ± 3.46 vs C+T: 43.04 ± 3.3 vs P: 38.58 ± 3.86 Sprint 6 peak power (W): C: 701.96 ± 154.62 vs T: 730.26 ± 153.31 vs C+T: 749.02 ± 155.74 vs P: 688.76 ± 147.55 Sprint 6 mean power (W): C: 301 ± 96.01 vs T: 335.66 ± 102.1 vs C+T: 352.82 ± 114.29 vs P: 288.11 ± 103.49 B[la] (mmol/L): C: 10.62 ± 2.1 vs T: 10.29 ± 2.22 vs C+T: 12.31 ± 2.54 vs P: 9.87 ± 1.97 HR (bpm): C: 185.59 ± 3.6 vs T: 178.68 ± 5.41 vs C+T: 180.49 ± 3.6 vs P: 188.59 ± 6.3 RPE: C: 16.88 ± 1.15 vs T: 18.56 ± 1.03 vs C+T: 18.5 ± 1.10 vs P: 18.44 ± 1.15	Yes only with C+T vs P in B[la]↑, C vs P/T/C+T in RPE and reaction time of ICR↓, C vs P/T in reaction time of CR↓ C/C+T vs P in TTE, C/T/C+T vs P on CMJ↑
Ozan et al. [17] RDB Crossover	30-s Wingate test (WanT); Balance test; Illinois agility test; Stroop test	20 males	Elite boxer (training 11.12 ± 1.12 years)	22.1 ± 1.4; 75.5 ± 9.0	C: 6 mg/kg T: 3 g C+T: 6 mg/kg +3 g P: 300 mg maltodextrin; Capsule; 1 h before	72 ± 0.5 h	Peak power of WanT (W): C: 687.66 ± 125.38 vs T: 653.64 ± 116.8 vs C+T: 726.20 ± 144.43 vs P: 633.02 ± 117.79 Mean power of WanT (W): C: 469.52 ± 84.18 vs T: 459.71 ± 87.74 vs C+T: 483.35 ± 87.20 vs P: 448.72 ± 82.89 Balance: Static both leg: C: 591.10 ± 278.98 vs T: 603.10 ± 297.04 vs C+T: 515.80 ± 225.55 vs P: 663.50 ± 297.33 Balance: Dynamic both leg: C: 1119.30 ± 256.32 vs T: 1128.40 ± 227.62 vs C+T: 1043.90 ± 245.62 vs P: 1252.30 ± 275.44 Agility (s): C: 16.95 ± 0.73 vs T: 16.75 ± 0.47 vs C+T: 16.44 ± 0.52 vs P: 18.79 ± 0.80 B[la] (mmol/L): C: 9.30 ± 1.49 vs T: 9.10 ± 1.09 vs C+T: 8.30 ± 1.33 vs P: 10.30 ± 1.16 RPE: C: 17.20 ± 1.13 vs T: 17.40 ± 0.84 vs C+T: 16.60 ± 0.69 vs P: 18.10 ± 1.42	Yes only with C/T/C+T vs P in reaction time of NR and ICR, error rate of ICR, agility test↓, C+T vs P in balance↓, C/T/C+T vs P, C+T vs T, in peak power↑, T/C+T vs P, C+T vs T, C vs T in mean power↑
Warnock et al. [42] RSB Crossover	3 × 30s Wingate tests	7 males	University trained team sports players	20.8 ± 0.9; 86.3 ± 10.2	C: 5 mg/kg T: 50 mg/kg C+T: 5 mg/kg +50 mg/kg P: 5 mg/kg maltodextrin; Capsule; 1 h before	48 h	Mean peak power (W): C: 825.13 ± 128.8 vs T:850.2 ± 81.56 vs C+T: 828.27 ± 125.66 vs P:734.12 ± 128.63 Peak power (W): C: 927.57 ± 181.07 vs T: 945.45 ± 115.71 vs C+T: 934.16 ± 121.81 vs P: 809.92 ± 178.51 Mean power (W): C: 595.85 ± 72.61 vs T: 612.5 ± 81.25 vs C+T: 600 ± 85.06 vs P: 558.33 ± 79.17 Inter-sprint fatigue (%):C: 14.89 ± 7.47 vs T: 11.13 ± 1.59 vs C+T: 15.4 ± 5.81 vs P: 11.57 ± 4.66 Intra-sprint fatigue (%): C: 65.38 ± 11.43 vs T: 74.18 ± 3.07 vs C+T: 62.75 ± 9.23 vs P: 64.73 ± 9.01 Heart rate(bpm): C: 164.9 ± 26.4 vs T: 161.5 ± 29.9 vs C+T: 158.2 ± 25.1 vs P: 159.6 ± 26.1 MAP (mmHg): C: 94.4 ± 19 vs T: 87.3 ± 5.1 vs C+T: 88.5 ± 6.1 vs P: 88.4 ± 9.7 B[La] (mmol/L): C: 9.7 ± 1.9 vs T: 8.6 ± 1.7 vs C+T: 8.8 ± 1.8 vs P: 8.9 ± 1.6 RPE: C: 17 ± 1 vs T: 17 ± 1 vs C+T: 17 ± 1 vs P: 18 ± 1	N/A
Yu et al. [43] RSB Crossover	Cycling TTE and CMJ under hot and humid conditions	12 males	University students majoring in sports took (training 3.04 ± 1.7 years)	23.8 ± 2.4; 75.7 ± 7.5	C: 5 mg/kg T: 50 mg/kg C+T: 5 mg/kg +50 mg/kg PLA: 5 mg/kg maltodextrin; Capsule; 1 h before	7 days	TTE (min): C: 21.8 ± 2.45 vs T: 23.89 ± 2.89 vs C+T: 21.72 ± 2.9 vs P: 20.48 ± 2.79 First CMJ peak power (W): C: 4244.81 ± 337.71 vs T: 4036.91 ± 324.69 vs C+T: 3916.02 ± 325.09 vs P: 3826.48 ± 355.54 First CMJ mean power (W): C: 4089.01 ± 354.72 vs T: 3919.05 ± 326.67 vs C+T: 3817.88 ± 320.5 vs P: 3688.90 ± 363.39 Heart rate (bpm): C: 178.42 ± 7.71 vs T: 178.25 ± 7.54 vs C+T: 171.76 ± 7.02 vs P:173.34 ± 4.9 B[La] (mmol/L): C: 10.01 ± 1.49 vs T: 7.71 ± 1.10 vs C+T: 10.22 ± 1.71 vs P: 8.71 ± 1.03 RPE: C: 18.4 ± 0.82 vs T: 18.74 ± 0.76 vs C+T: 18.52 ± 0.76 vs P: 19.15 ± 0.82	Yes only with C vs T/C+T/P in CMJ, T vs P on TTE↑, T vs C/C+T in B[La]↓

Abbreviation: bpm: Beats per minutes; B[La]: Blood lactate; C: Caffeine; T: Taurine; C+T: Caffeine + taurine; CMJ: Countermovement jump; MD: Manipulate diathermy; MIST-VR: Minimally invasive surgical trainer – Virtual reality; N/A: Not applicable; P: Placebo; RDB: Randomized double-blind trial; RSB: Randomized single-blind trial; RPE: Rating of perceived exertion; SD: Stretch diathermy; TTE: Time to exhaustion.

**Table 2.** Results and characteristics of individual studies on cognitive function or focus on physiological responses.

Study and design	Test protocol	Sample	Training status	Age (y); Weight (kg)	Dosage; Form; Timing	Washout period	Outcomes	Statistical significance (*p* < 0.05)
Aggarwal et al. [39] RSB Crossover	PVT, MAPc, Stroop task, WCST，SSS	18 NS	Healthy untrained students	>18/ N/A	C: 150 mg C+T: 150 mg +2 g PLA: vitamin C and calcium capsule (NS); Capsule; 1 h before	Min 48 h	PVT time (ms): C: 307.25 ± 51.11 vs C+T: 325.25 ± 62.96 vs P: 427.75 ± 243.7 Stroop task time (s): C: 21.95 ± 10.52 vs C+T: 24.23 ± 8.22 vs P: 28.1 ± 16.44 WCST: C: 13.75 ± 5.19 vs C+T: 15 ± 4.44 vs P: 17.25 ± 6.67 MAPc time (s): C: 206.5 ± 71.11 vs C+T: 190.75 ± 68.89 vs P: 205 ± 84.44 SSS: C: 3 ± 1.48 vs C+T: 3.5 ± 1.48 vs P: 5.75 ± 0.74	N/A
Bichler et al. [12] RDB Crossover	Short term explicit memory test	8 females and 6 males	Healthy unspecified student	20.5 ± 1.5; N/A	C+ T: 80 mg +1000 mg PLA: candy pill (NS); Capsule; 45 min before	24–240 h	Correct answer of memory test: C+T = P HR (bpm): C+T: ↓ from baseline to ending，P: No change	Yes only with C+T HR from baseline to ending ↓
Çalışkan [13] RSB Parallel	EEG	16 females and 40 males (4 groups, each 16)	Healthy untrained students	20.1 ± 2; 69.6 ± 14.6	C: 200 mg T: 2500 mg C+T: 200 mg +2500 mg P: consumed the same amount of water within 5 min; Capsule; 30 min and 60 min before	NS	30 min and 60 min heart rate (bpm): C: 80.86 ± 8.8 and 89.57 ± 8.24 vs T: 82.36 ± 12.84 and 82.64 ± 12.68 vs C+T: 85.93 ± 9.32 and 87.43 ± 8.48 vs P: 77.24 ± 10.04 and 78.49 ± 12.04	Yes only with 60 min C vs 30 min C in heart rate ↑
Giles et al. [40] RDB Crossover	ANT, NB, CRT, SRT	16 females and 8 males	Healthy unspecified students	20.1 ± 1.9; N/A	C: 200 mg T: 2000 mg C+T: 200 mg +2000 mg P: NS; Capsule; 30 min and 60 min before	3 days	ANT alerting, orienting and executive function: C: 44.71 ± 25.13, 31.46 ± 10.06 and 81.71 ± 21.41 vs T: 44.92 ± 29.88, 30.96 ± 31.46 and 86.42 ± 27.01 vs C+T: 38.79 ± 27.76, 31.42 ± 12.4 and 87.67 ± 31.81 vs P: 39.00 ± 19.27, 33.04 ± 14.00 and 93.04 ± 25.65 SRT reaction time (ms):C: 158.37 ± 14.32 vs T: 159.58 ± 17.14 vs C+T: 154.27 ± 13.61 vs P: 160.12 ± 19.96 CRT accuracy and reaction time (ms): C: 0.96 ± 0.01 and 390.76 ± 12.90 vs T: 0.96 ± 0.00 and 393.37 ± 13.62 vs C+T: 0.96 ± 0.00 and 386.21 ± 14.76 vs P: 0.95 ± 0.01 and 409.32 ± 20.42 Verbal NB reaction time (ms): C: 700.34 ± 37.57 vs T: 701.21 ± 42.04 vs C+T: 679.67 ± 36.57 vs P: 731.09 ± 35.38 Object NB sensitivity: C: 2.44 ± 0.22 vs T: 2.30 ± 0.26 vs C+T: 2.71 ± 0.16 vs P: 2.33 ± 0.30 Spatial NB Hit rat, reaction time (ms) and sensitivity: C: 0.81 ± 0.03, 709.81 ± 42.57 and 2.52 ± 0.23 vs T: 0.82 ± 0.04, 677.18 ± 41.47 and 2.45 ± 0.25 vs C+T: 0.82 ± 0.03, 689.65 ± 41.65 and 2.70 ± 0.17 vs P: 0.85 ± 0.03, 706.72 ± 39.98 and 2.49 ± 0.28	Yes only with C/T vs P in NB verbal and object↓, C vs P in spatial↑ C vs P in reaction time of SRT and CRT ↓，T vs P in accuracy of CRT↑
Kammerer et al. [20] RDB Crossover	FAT, DIGITS	14 males	Healthy trained soldiers	20 ± 1; 68 ± 7.2	C: 80 mg T: 1000 mg C+T: 80 mg +1000 mg PLA: Similar flavor carbonated-based water; Beverage; 45 min before	48–72 h	FAT: C: 19.8 ± 4.4 vs T: 19.1 ± 4.7 vs C+T: 20.6 ± 4.5 vs P: 19.9 ± 5.9 DIGITS: C: 10.6 ± 2.9 vs T: 11.2 ± 2.7 vs C+T: 11.0 ± 2.8 vs P: 10.9 ± 3.1	No
Liu & Rong [41] RDB Crossover	Stroop test	16 males	University football players (training over 5 times/week)	23.7 ± 2.2; 75.0 ± 7.8	C: 5 mg/kg T: 50 mg/kg C+T: 5 mg/kg +50 mg/kg PLA: 5 mg/kg maltodextrin; Capsule; 1 h before	3 days	CR and ICR reaction time (ms): C: 505.24 ± 36.32 and 531.41 ± 36.89 vs T: 556.38 ± 34.47 and 587.66 ± 28.86 vs C+T: 530.71 ± 37.80 and 575.22 ± 49.40 vs P: 561.29 ± 29.41 and 581.84 ± 27 CR and ICR accuracy rate (%): C: 0.933 ± 0.027 and 0.869 ± 0.038 vs T: 0.934 ± 0.035 and 0.909 ± 0.055 vs C+T: 0.928 ± 0.027 and 0.863 ± 0.056 vs P: 0.917 ± 0.037 and 0.883 ± 0.048	Yes only with C vs P/T/C+T in reaction time of ICR↓, C vs P/T in reaction time of CR↓
Ozan et al. [17] RDB Crossover	Stroop test	20 males	Elite boxer (training 11.12 ± 1.12 years)	22.1 ± 1.4; 75.5 ± 9.0	C: 6 mg/kg T: 3 g C+T: 6 mg/kg +3 g P: 300 mg maltodextrin; Capsule; 1 h before	72 ± 0.5 h	NR, CR and ICR reaction time (ms): C: 723.53 ± 135.29, 588.24 ± 105.88 and 729.41 ± 114.71 vs T: 714.71 ± 105.88, 585.29 ± 105.89 and 735.29 ± 111.77 vs C+T: 579.41 ± 117.65, 544.12 ± 108.82 and 652.94 ± 138.24 vs P: 829.41 ± 144.12, 702.94 ± 147.06 and 938.24 ± 100 NR, CR and ICR error rate (%): C: 5.25 ± 1.16, 4.17 ± 1.76 and 5.93 ± 2 vs T: 5.05 ± 1.29, 4.37 ± 1.16 and 6.17 ± 1.93 vs C+T: 4.54 ± 2.17, 3.25 ± 2.68 and 4.24 ± 1.86 vs P: 7.76 ± 2.75, 4.68 ± 2.2 and 10.03 ± 2.44	Yes only with C/T/C+T vs P in reaction time of NR and ICR, error rate of ICR↓
Peacock et al. [18] RDB Crossover	Visual oddball task and stimulus degradation task (3 types: intact, high, low degradation)	19 females	Healthy untrained undergrad	20.8 ± 0.9; N/A	C: 80 mg T:1000 mg C+T: 80 mg +1000 mg P: matched weight cornflour; Capsule; 45 min before	2–7 days	Reaction time (ms): Visual oddball: C: 399 ± 44 vs T: 403 ± 44 vs C+T: 389 ± 33 vs P: 406 ± 40, stimulus degradation: Intact: C: 604 ± 15 vs T: 625 ± 17 vs C+T: 623 ± 22 vs P: 642 ± 12 Low: C: 617 ± 15 vs T: 637 ± 16 vs C+T: 641 ± 20 vs P: 655 ± 14 High: C: 646 ± 15 vs T: 670 ± 18 vs C+T: 663 ± 18 vs P: 688 ± 14	Yes only with C vs P in stimulus degradation↓

Abbreviation: ANT: Attention network test; bpm: Beats per minutes; C: Caffeine; T: Taurine; C+T: Caffeine + taurine; CRT: Choice reaction task; DIGITS: Digit span WAIS subtest; EEG: Electrocardiogram; FAT: Focused attention test; NB: N-back task; N/A: Not applicable; P: Placebo; RDB: Randomized double-blind trial; RSB: Randomized single-blind trial; RRT: Reaction time task; SRT: Sample reaction task.

### 3.3. Primary analysis

#### 3.3.1. Physical capacity

**Figure 2.**
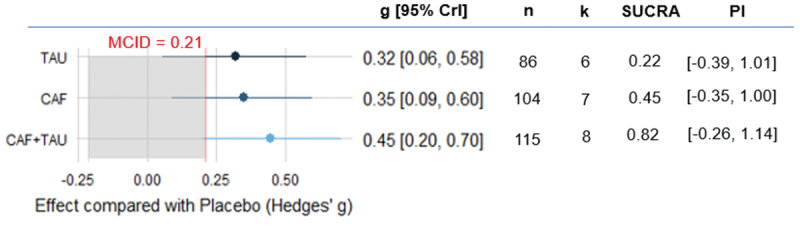
Estimated effects of caffeine, taurine, and their combination on physical capacity.

CAF: Caffeine; CAF+TAU: Caffeine and taurine co-supplementation; CrI: Credible intervals; k: Number of included studies; MCID: Minimal clinically important difference; n: Number of included participants; PI: Prediction interval; SUCRA: Surface under the cumulative ranking curve; TAU: taurine.

Our meta-analysis results showed that CAF (k = 7, g = 0.35, 95% CrI [0.09, 0.60], PI [−0.35, 1.00]), TAU (k = 6, g = 0.32, 95% CrI [0.06, 0.58], PI [−0.39, 1.01]), and CAF+TAU (k = 8, g = 0.45, 95% CrI [0.20, 0.70], PI [−0.26, 1.14]) each demonstrated a credible positive effect on physical capacity. However, no substantial differences were observed between the interventions based on pairwise posterior comparisons. All of these treatments demonstrated effect sizes exceeding the MCID, defined as an absolute g value of 0.21. When constructing the SUCRA, CAF+TAU was the treatment most likely to perform best (Figure 2).

#### 3.3.2. Cognitive function

**Figure 3.**
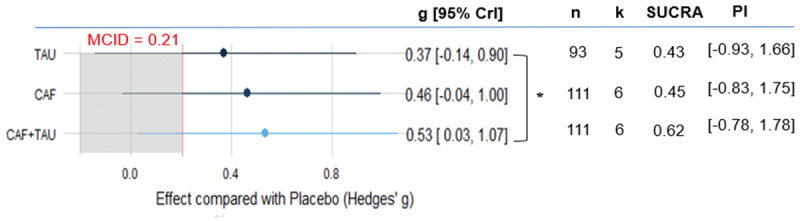
Estimated effects of caffeine, taurine, and their combination on cognitive function.

CAF: Caffeine; CAF+TAU: Caffeine and taurine co-supplementation; CrI: Credible intervals; k: Number of included studies; MCID: Minimal clinically important difference; n: Number of included participants; PI: Prediction interval; SUCRA: Surface under the cumulative ranking curve; TAU: taurine; *: Indicates a credible difference between groups, as the 95% HPD interval did not include zero.

For cognitive ability, our meta-analysis results showed that although all of these treatments are significantly stronger than the MCID, only CAF+TAU demonstrated a credible positive effect (k = 6, g = 0.53, 95% CrI [0.03, 1.07], PI [−0.78, 1.78]), as its 95% credible interval excluded zero. Furthermore, CAF+TAU showed a credibly greater improvement in cognition compared to TAU, as the 95% HPD interval of the estimated difference did not include zero. At the same time, CAF+TAU has the highest SUCRA value (0.62), indicating that this combination treatment may be the most effective option among the three interventions (Figure 3). For more details on the differences between the groups, please refer to Electronic Supplementary Material Appendix S3.

#### 3.3.3. RPE

**Figure 4.**
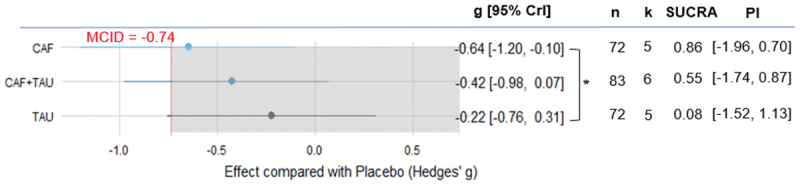
Estimated effects of caffeine, taurine, and their combination on RPE.

CAF: Caffeine; CAF+TAU: Caffeine and taurine co-supplementation; CrI: Credible intervals; k: Number of included studies; MCID: Minimal clinically important difference; n: Number of included participants; PI: Prediction interval; SUCRA: Surface under the cumulative ranking curve; TAU: taurine; *: Indicates a credible difference between groups, as the 95% HPD interval did not include zero.

Our meta-analysis results showed that only CAF demonstrated a credible effect in reducing RPE compared with placebo (k = 5, g = −0.64, 95% CrI [−1.20, −0.10], PI [−1.96, 0.70]), and it yielded the highest SUCRA value (0.86). CAF also showed a credibly stronger effect in reducing RPE compared to TAU, as indicated by the corresponding 95% HPD interval excluding zero. Both CAF and CAF+TAU exceeded the MCID (g = −0.74), while TAU’s effect size was close to the threshold. TAU had the lowest SUCRA value (0.08), suggesting that this treatment may have the least impact on reducing RPE (Figure 4). For more details on the differences between the groups, please refer to Electronic Supplementary Material Appendix S3.

#### 3.3.4. HR

**Figure 5.**
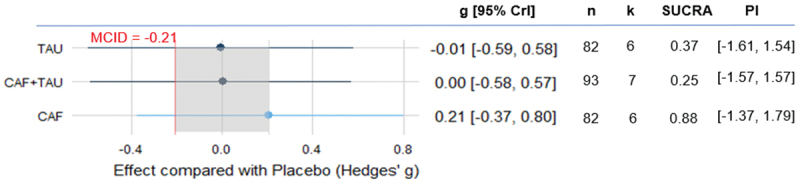
Estimated effects of caffeine, taurine, and their combination on HR.

CAF: Caffeine; CAF+TAU: Caffeine and taurine co-supplementation; CrI: Credible intervals; k: Number of included studies; MCID: Minimal clinically important difference; n: Number of included participants; PI: Prediction interval; SUCRA: Surface under the cumulative ranking curve; TAU: taurine.

Our meta-analysis results indicated that none of the three interventions (TAU, CAF+TAU, and CAF) demonstrated a credible effect on HR compared to placebo, as all 95% credible intervals included zero. Although the estimated effect sizes for all interventions exceeded the MCID threshold of −0.21, the posterior uncertainty was substantial. CAF alone had the highest SUCRA value (0.88), suggesting it may be the most likely option to increase HR. However, its effect size remained small and uncertain (k = 6, g = 0.21, 95% CrI [−0.37, 0.80], PI [−1.37, 1.79]). Additionally, TAU and CAF+TAU showed minimal overall changes in HR compared to placebo (Figure 5). Overall, these interventions may have limited clinical benefits.

#### 3.3.5. B[la]

**Figure 6.**
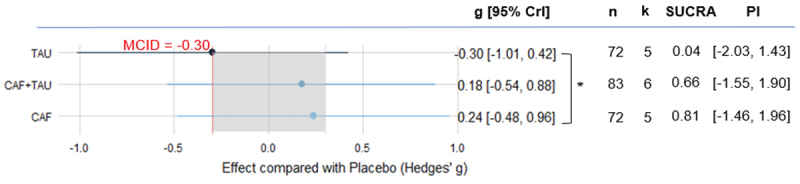
Estimated effects of caffeine, taurine, and their combination on B[la].

CAF: Caffeine; CAF+TAU: Caffeine and taurine co-supplementation; CrI: Credible intervals; k: Number of included studies; MCID: Minimal clinically important difference; n: Number of included participants; PI: Prediction interval; SUCRA: Surface under the cumulative ranking curve; TAU: taurine; *: Indicates a credible difference between groups, as the 95% HPD interval did not include zero.

For B[la], our meta-analysis results showed that the MCID was defined as −0.30, and none of the three interventions (TAU, CAF+TAU, and CAF) demonstrated a credible effect compared with placebo, as all 95% credible intervals included zero. CAF showed the largest estimated increase in B[la], with the highest SUCRA value (0.81) and a positive effect size (g = 0.24), followed by CAF+TAU (SUCRA = 0.66, g = 0.18), while TAU exhibited a negative effect (SUCRA = 0.04, g = −0.30) (Figure 6). Notably, pairwise Bayesian comparisons indicated that both CAF and CAF+TAU were credibly higher than TAU, as the corresponding 95% HPD intervals of their differences excluded zero. These findings suggest that, although none of the interventions showed a credible effect versus placebo, TAU was associated with lower lactate levels compared with CAF or CAF+TAU, based on its effect estimate and SUCRA ranking. For more details on the differences between the groups, please refer to Electronic Supplementary Material Appendix S3.

### 3.4. Moderator analysis

We conducted moderator analysis to separate the effects of CAF, TAU, and CAF+TAU on anaerobic and aerobic capacity as well as reaction time and other cognitive abilities.

#### 3.4.1. Physical capacity type — aerobic capacity/anaerobic capacity

**Figure 7.**
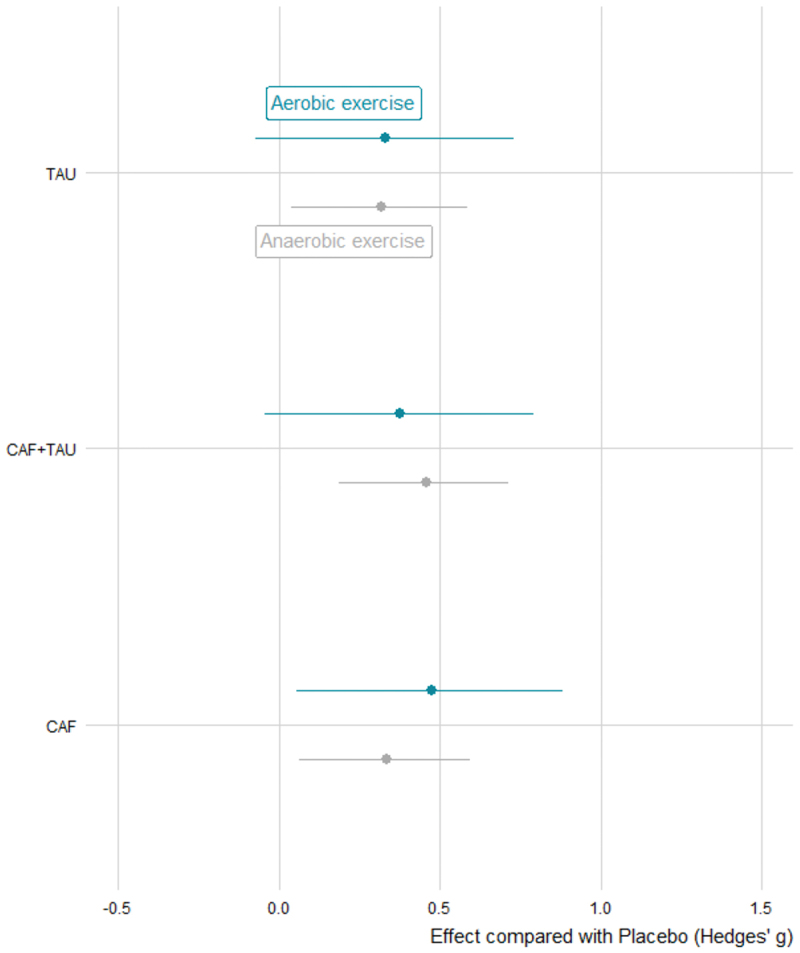
Estimated effects of caffeine, taurine, and their combination on aerobic and anaerobic capacity.

CAF: Caffeine; CAF+TAU: Caffeine and taurine co-supplementation; TAU: taurine.

Our meta-analysis results showed that CAF demonstrated consistent positive effects on both anaerobic and aerobic capacity compared to placebo, as reflected by its posterior estimates and relatively narrow credible intervals (Figure 7). In contrast, the effects of TAU and CAF+TAU on aerobic capacity were less certain, indicated by wider 95% CrIs. No credible differences were observed between aerobic and anaerobic outcomes under the same treatment condition, as all corresponding paired HPD intervals included zero. However, under anaerobic conditions, the estimated effect of CAF+TAU (g = 0.46, 95% CrI [0.19, 0.71]) showed only slight overlap of the HPD interval with zero, compared to TAU (g = 0.32, 95% CrI [0.04, 0.58]) and CAF (g = 0.33, 95% CrI [0.06, 0.60]), suggesting a potentially clinically meaningful difference between treatments despite overlapping posterior uncertainty. For more details on the differences between the groups, please refer to Electronic Supplementary Material Appendix S3.

#### 3.4.2. Cognitive function type — reaction time/other cognitive capacity

**Figure 8.**
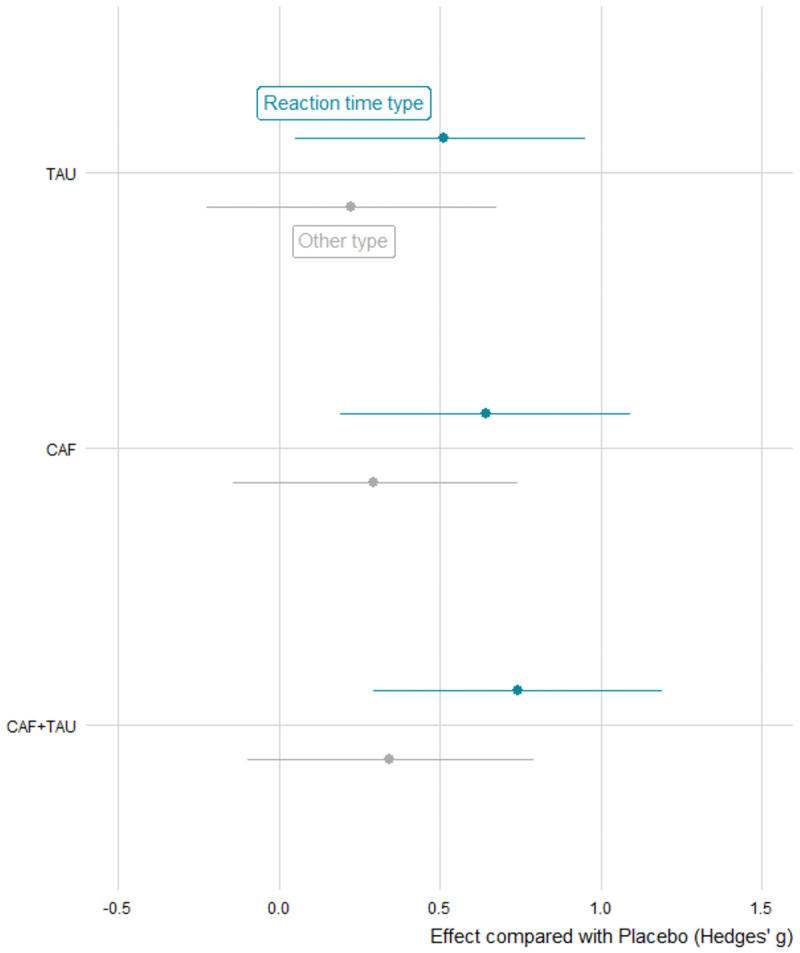
Estimated effects of caffeine, taurine, and their combination on reaction time and other cognitive functions.

CAF: Caffeine; CAF+TAU: Caffeine and taurine co-supplementation; TAU: taurine.

Our meta-analysis revealed that CAF, TAU, and CAF+TAU appeared more effective in enhancing reaction time compared to other cognitive domains. Among these, CAF+TAU (g = 0.75, 95% CrI [0.29, 1.18]) demonstrated the greatest estimated improvement (Figure 8). However, the comparison between CAF+TAU and CAF involved some posterior uncertainty, as the corresponding HPD interval slightly included zero. Likewise, CAF showed a numerically greater effect than TAU in improving reaction time, although the 95% HPD interval marginally overlapped with zero, indicating that the difference was not credibly supported. For more details on the differences between the groups, please refer to Electronic Supplementary Material Appendix S3.

### 3.5. Risk of bias and quality of methods

The risk of bias for each study is depicted in Electronic Supplementary Material Appendix S4. While most studies indicated low risk in key areas, several studies showed methodological limitations. Specifically, the randomization process was rated as some concerns in five studies [6,19,20,40,42], mainly due to inadequate reporting of the allocation method. One study was considered to be at high risk for outcome selection due to suspected selective reporting and lack of protocol transparency [40]. In addition, approximately 10% of studies had concerns about outcome measurement, mainly due to potential detection bias in unblinded assessment. Notably, Giles et al. [40] study was the only one rated as having a high overall risk of bias. Overall, while the majority of studies were rated as “low risk,” a moderate proportion (~40%) were classified as having some concerns.

There was evidence of publication bias for CAF, TAU, and CAF+TAU estimates of physical capacity and cognitive ability compared with placebo (funnel plot asymmetry and Egger’s test *p* < 0.01), although this was not sufficient to offset the effect (S-value = not possible). In contrast, no publication bias was found for CAF, TAU, and CAF+TAU estimates of RPE, HR, and B[la], as indicated by non-significant Egger’s test results (*p* > 0.1). However, the S-value results varied across these outcomes: for RPE and B[la], the S-value was either “not possible” or close to 1, suggesting limited influence of publication bias, whereas for HR, the S-value was notably higher for CAF (16.76) compared to CAF+TAU (1.98) and TAU (1.13), indicating potential differences in bias susceptibility across treatments. Detailed results are provided in Electronic Supplementary Material Appendix S5.

Electronic Supplementary Material Appendix S6 presents details of the QualSyst evaluation, all included studies were classified as moderate to strong in terms of methodology, potentially increasing the confidence of conclusions.

### 3.6. Sensitivity analysis

Under different indicators, direct and indirect estimates for CAF, TAU, and CAF+TAU showed no statistically significant differences across all paired comparisons (*p* > 0.05), supporting the consistency assumption of the model. However, given the simple network structure, indirect estimates in some comparisons were derived from a single pathway, leading to substantial numerical discrepancies between direct and indirect estimates, which may indicate potential instability. Additionally, since the number of included studies was relatively small (k = 5–8), the statistical power for detecting inconsistency was limited. As a result, even if true inconsistency exists, the test may lack sensitivity, potentially leading to non-significant p-values despite meaningful differences in estimates.For the details, please refer to Electronic Supplementary Material Appendix S7.

## 4. Discussion

The aim of this systematic review and meta-analysis was to explore the effects of co-supplementation of CAF and TAU on physical capacity, cognitive function, and physiological parameters in comparison with the intake of these substances alone.

### 4.1. Physical capacity

Our meta-analysis indicated that CAF, TAU, and CAF+TAU improved physical capacity, with CAF+TAU showing the most credible effects. Further analysis of the effects of different intervention methods on aerobic and anaerobic exercise capacity found that CAF+TAU maintained the best effect on anaerobic capacity, while showing uncertainty in the improvement of aerobic capacity.

This discrepancy may be attributed to the synergistic effects of CAF and TAU under anaerobic conditions, where CAF enhances central nervous system (CNS) excitability and promotes glycolysis, thereby supporting high-intensity power output [3]. TAU complements this by stabilizing intracellular calcium homeostasis, improving muscle contractility, and reducing oxidative stress [44]. Their combined effects may further facilitate sarcoplasmic reticulum calcium release and Na+/K±ATPase activity, ultimately enhancing skeletal muscle force production and increasing myofilament sensitivity [7,45,46]. This is supported by two studies using the Wingate test, in which CAF+TAU resulted in higher power output compared to either supplement alone [6,17].

However, the potential antagonistic effect of TAU on CAF-induced central stimulation and lipid oxidation during aerobic exercise may attenuate the expected endurance benefits of CAF+TAU, contributing to the observed uncertainty [44,47].

Additionally, variations in aerobic capacity testing protocols and participant characteristics across the included studies may have influenced the results. For example, Yu et al. [43] reported that TAU alone significantly improved cycling time-to-exhaustion (TTE) performance in moderately trained college students under hot and humid conditions (*p* < 0.05), attributing this to its thermoregulatory properties, including enhanced mitochondrial efficiency, reduced glycolytic dependence, and improved heat dissipation [48,49]. This was corroborated by a lower core temperature (CT) in the TAU group, whereas the CT of the CAF+TAU group more closely resembled that of the CAF group, suggesting that the thermogenic effect of CAF may counteract TAU cooling benefits [50]. Similarly, Liu et al. [41] observed that CAF+TAU improved cycling TTE performance in trained college football players under hypoxic conditions, although the underlying mechanism remains unclear. In contrast, [20] found no improvement in endurance performance with any supplementation during a graded treadmill test involving soldier participants. This null finding may reflect their uniformly high baseline fitness levels, structured training routines, and standardized diets, which could have constrained the scope for measurable enhancement in physiological or cognitive outcomes, indicating a potential ceiling effect. These findings suggest that the ergogenic potential of CAF+TAU on endurance performance is context-dependent, potentially modulated by both environmental challenges and interindividual factors such as habitual training status and fitness baseline.

### 4.2. RPE

Our results showed that CAF was associated with the most credible reduction in RPE, suggesting its potential to lower perceived exertion while supporting exercise performance. Mechanistically, CAF reduces RPE mainly through adenosine receptor antagonism of A1 and A2A receptors, thereby reducing central fatigue, enhancing dopaminergic and noradrenergic activity, and improving arousal and motivation [3]. In addition, CAF optimizes neuromuscular efficiency by enhancing motor unit recruitment and transmission, while shifting substrate utilization toward increased lipid oxidation and glycogen retention, thereby delaying metabolic fatigue and reducing the perception of exertion during prolonged exercise [51,52]. CAF may also reduce pain perception through the endogenous opioid system, further contributing to the reduction of RPE [53].

In contrast, TAU had a weaker effect, which may be due to its indirect physiological benefits rather than direct sensory modulation, making it less effective than CAF in reducing RPE. Yu et al. [43] demonstrated that TAU alone was not effective in reducing RPE, but it may still provide long-term advantages for fatigue resistance, especially under heat-challenged conditions.

CAF+TAU provided a modest reduction in RPE, although it did not exceed the effect of CAF alone. This may be due to TAU attenuating CAF-induced excitability by modulating neurotransmitter release and reducing CNS overactivation, which may inhibit the stimulatory effects of CAF on arousal and perceived effort. Nonetheless, given the superior performance of CAF+TAU on anaerobic capacity, it appears to offer a balanced approach for athletes seeking to optimize high-intensity performance while maintaining lower perceived effort.

### 4.3. B[la]

As a practical marker of anaerobic system engagement, B[la] reflects the dynamic balance between muscular lactate production and systemic clearance [54,55], and provides insight into how CAF and TAU modulate energy metabolism during exercise-induced stress. Our findings indicate that while CAF enhances physical capacity and is the most effective in reducing RPE, it is also associated with a moderate increase in B[la] (g = 0.24, SUCRA = 0.81). This likely reflects a greater reliance on anaerobic metabolism during high-intensity efforts, driven by CAF’s stimulatory effects on catecholamine release, upregulated glycolytic flux, and increased energy turnover under supramaximal conditions [51], which collectively supports its glycolytic-promoting metabolic profile.

In contrast, the reduction in B[la] associated with TAU (g = −0.30, SUCRA = 0.04) may reflect enhanced lactate clearance or reduced lactate production during high-intensity exercise. This effect is potentially attributed to TAU’s capacity to enhance mitochondrial respiration and ATP synthesis, promoting aerobic energy pathways and reducing reliance on anaerobic glycolysis [48,49]. Additionally, TAU has been shown to upregulate monocarboxylate transporters (MCT1 and MCT4), facilitating lactate efflux from muscle and enhancing its oxidation in metabolically active tissues such as the heart and liver [48], which may provide a further mechanism for this observed effect.

The intermediate effect of CAF+TAU on B[la] (g = 0.18, SUCRA = 0.66) suggests a potential balancing interaction between CAF’s glycolytic stimulation and TAU’s facilitative role in lactate turnover and mitochondrial support. However, this effect was not statistically robust, and its variability across studies may stem from differences in exercise modality, supplementation timing, or individual metabolic profiles. While CAF+TAU has demonstrated superior effects on anaerobic performance in our analysis, its role in modulating exercise-related metabolic responses remains inconclusive. Further research is needed to clarify whether TAU complements CAF’s effects without compromising its ergogenic benefits.

### 4.4. HR

Our results suggest that CAF exhibited the greatest potential for increasing HR among the three interventions, though this effect remains uncertain. Prior research has demonstrated that CAF intake induces vasoconstriction and stimulates norepinephrine release, leading to increases in both blood pressure and HR [56,57]. In contrast, TAU and CAF+TAU showed minimal overall changes in HR compared to placebo, which may be attributable to TAU’s ability to modulate cardiovascular responses by inhibiting norepinephrine release, enhancing norepinephrine turnover, and attenuating angiotensin II-mediated vasoconstriction [58].

Notably, no credible differences were observed in HR between CAF, TAU, CAF+TAU, and placebo. A plausible explanation is that HR measurements in the included studies were primarily obtained during exercise performance, where exercise intensity may have reached a physiological threshold sufficient to stabilize HR or push it toward its peak range, thereby minimizing differences between intervention and placebo conditions. This aligns with prior findings that at higher exercise intensities, sympathetic activation and cardiac autonomic regulation may dominate HR responses, limiting the capacity of exogenous stimulants to elicit further increases [3]. Overall, these findings suggest that CAF, TAU, and CAF+TAU have limited ergogenic effects on HR modulation during exercise, reinforcing the importance of exercise intensity and interindividual variability in shaping cardiovascular responses.

In resting conditions, Çalışkan et al. [13] reported CAF significantly increased HR and concurrently elevated nonlinear cardiovascular parameters associated with pathological dynamics, including the largest Lyapunov exponent and approximate entropy. Although CAF+TAU exhibited a similar increasing trend in these indicators, only the largest Lyapunov exponent reached statistical significance, whereas TAU alone did not elicit significant changes [13]. These findings suggest that CAF has the most pronounced impact on cardiovascular complexity, potentially increasing cardiac system instability in young individuals. However, the presence of TAU may mitigate some of the adverse effects associated with CAF, highlighting its potential cardioprotective role in counteracting excessive sympathetic activation and promoting autonomic balance. Further research is warranted to elucidate the mechanisms underlying these interactions and their implications for cardiovascular health.

### 4.5. Cognitive function

Our meta-analysis showed that CAF, TAU, and CAF+TAU all improved cognitive performance, with CAF+TAU showing the largest effect and being the only intervention with a credible interval excluding zero (g = 0.53, 95% CrI [0.03,1.07]). This suggests that CAF+TAU may offer the most reliable cognitive benefits. SUCRA rankings (CAF+TAU: 0.62; CAF: 0.45; TAU: 0.43) further supported its superiority.

The mechanistic basis for these findings lies in the distinct roles of CAF and TAU in cognitive function. CAF enhances reaction time by blocking adenosine receptors, which reduces mental fatigue and increases catecholamine release (dopamine and norepinephrine), thereby improving motor responses and cognitive processing speed [57,59]. In contrast, TAU does not directly stimulate cognitive function but rather modulates neurotransmitter turnover and calcium homeostasis, contributing to neuroprotection rather than acute cognitive enhancement [58,60]. The synergistic effect between the two compounds in CAF+TAU may optimize cognitive function by providing both immediate stimulation and long-term neural stability, preventing overstimulation while sustaining cognitive performance [56].

When categorized by cognitive domain, CAF, TAU, and CAF+TAU were more effective in improving reaction time than other cognitive abilities. CAF+TAU maintained the largest effect, though its advantage over CAF alone was associated with some uncertainty, as indicated by HPD intervals slightly crossing zero. For other cognitive functions, all interventions exceeded the minimal clinically important difference (MCID = 0.21), but their effects were weaker compared to reaction time. The benefits of CAF were primarily linked to alertness and arousal, while its effects on higher-order cognitive functions, such as complex information processing, were more modest [52,61]. Meanwhile, TAU’s role appeared more supportive than stimulatory, likely due to its neuromodulatory and neuroprotective properties rather than direct enhancement of cognitive speed [49]. This suggests that the advantages of CAF+TAU are most evident in tasks that require rapid reaction times rather than sustained memory or executive function.

### 4.6. Dose dependency and timing consideration

The ergogenic effects of CAF and TAU co-ingestion appear to be highly dose-dependent, with dose balance emerging as a critical factor. An unbalanced ratio (e.g. too low a dose of CAF or too high a dose of TAU), may further enhance the palliative effect of TAU and diminish the stimulatory effect of CAF. According to the literature, energy drinks typically contain 40–325 mg of caffeine, comparable to 3–6 mg/kg in capsule form [62], and 71–3105 mg of TAU, with the most commonly used dose ranging from 1000 to 2000 mg [63]. The reviewed studies show that the commonly available CAF and TAU doses in energy drinks, such as Red Bull® (CAF: 80 mg, TAU: 1 g), are ineffective in enhancing motor or cognitive performance [12,18,20]. This contrasts with findings that the same doses of CAF and TAU in energy drinks can improve performance, suggesting that other ingredients may contribute synergistically, though further investigation is needed [52,64]. High-dose combinations (e.g. 5 mg/kg CAF and 50 mg/kg TAU) show greater promise for endurance and high-intensity tasks [41–43]. Additionally, the differing absorption peaks of CAF, typically between 15 minutes to 60 minutes with a mean of 30 minutes, and TAU, occurring around 1 to 2.5 hours with a mean of 1.5 hours, may limit their synergistic effects, emphasizing the need for careful consideration on co-supplementation timing in future studies [60,65].

### 4.7. Strength and limitations

This study represents the first systematic review and meta-analysis focusing exclusively on the two primary active ingredients in energy drinks, CAF and TAU, comprehensively evaluating their potential synergistic or antagonistic effects across exercise performance, cognitive function, and physiological responses. A key strength of this review is the use of an arm-based Bayesian network model, which effectively addresses challenges arising from multiple related effect sizes within the same study, enhancing the robustness and interpretability of the results. In addition, we provided the detailed explanations of our statistical procedures to enhance transparency, reproducibility, and readability of the methodology.

Nevertheless, several limitations must be acknowledged. First, the relatively small number of included studies increases the influence of individual study variability, which may compromise the precision and robustness of the model estimates, thereby affecting the generalizability and reliability of the conclusions. This also prevented further exploration by detailed grouping of sport type, athlete’s competition level, and different doses and timings of supplementation. Second, although multiple statistical approaches (e.g. S-value analysis, prediction intervals) were employed to assess result robustness, the effect sizes in this meta-analysis were derived from comparisons between experimental and placebo groups. As a result, direct assessments of within-study inconsistency between the experimental arms and placebo groups were not feasible. This limitation also precludes the application of the CINeMA (Confidence in Network Meta-Analysis) framework, making it impossible to directly quantify the credibility of each effect estimate. Notably, the prediction intervals for all treatments crossed the zero line, indicating the presence of between-study heterogeneity and limiting the certainty of predictions.

Despite these limitations, our methodologically rigorous approach, incorporating a Bayesian network meta-analysis framework and multiple robustness verification strategies, provides valuable insights into the combined effects of CAF and TAU, paving the way for further high-quality investigations.

### 4.8. Future research directions

Based on the findings of this meta-analysis, several research gaps warrant further investigation.

Firstly, the dose-dependent effects of CAF+TAU require further exploration to determine the optimal ratio that maximizes ergogenic benefits while minimizing potential antagonistic interactions. Given the distinct roles of CAF in stimulating the central nervous system and TAU in modulating neurotransmission, future studies should clarify whether different CAF-to-TAU ratios are more effective for endurance versus anaerobic tasks and whether excessive TAU dampens CAF’s stimulatory effects. Secondly, the context-specific effects of CAF+TAU remain unclear, as its efficacy appears to vary across environmental conditions (e.g. heat, hypoxia) and exercise modalities. While TAU has been shown to enhance thermoregulation, it remains unclear whether this effect influences its synergy with CAF during prolonged endurance tasks. Investigating the impact of training status, habitual caffeine consumption, and individual metabolic responses will also be critical in refining supplementation recommendations. Thirdly, the interplay between CAF and TAU in lactate regulation warrants further investigation. While CAF promotes glycolysis and is associated with greater lactate accumulation, TAU may facilitate lactate clearance by enhancing mitochondrial respiration and supporting the expression of lactate transporters. Considering that exercise-induced acidosis results primarily from hydrogen ion accumulation rather than lactate itself, future studies should examine whether TAU can help preserve acid – base balance and reduce fatigue while maintaining the anaerobic performance benefits associated with CAF. This question is particularly relevant for high-intensity, intermittent exercise contexts. Fourth, the timing and absorption kinetics of CAF and TAU should be optimized. Given that CAF reaches peak plasma concentration within about 30 minutes, whereas TAU takes about 1.5 hours, their simultaneous ingestion may limit potential synergy. Studies should assess whether staggered intake enhances cognitive and physical capacity by aligning peak absorption windows with the most metabolically demanding phases of exercise. Lastly, long-term safety and neuromodulatory effects of CAF+TAU supplementation remains underexplored. While CAF is well-documented for its acute performance-enhancing effects, chronic use has been associated with potential cardiovascular strain. TAU, on the other hand, has been suggested to modulate autonomic regulation and support neuroprotection, but its long-term implications in athletes and general populations remain unclear. Future research should assess the prolonged impact of CAF+TAU on cognitive resilience, autonomic balance, and cardiovascular health to determine its viability as a sustainable performance aid.

### 4.9. Practical implications

Based on the results of this systematic review and meta-analysis, athletes should consider using a combination of CAF+TAU to potentially improve athletic and cognitive performance, particularly in tasks that increase anaerobic capacity and reaction time.

## 5. Conclusion

Despite some inconsistencies in the field, this systematic review and network meta-analysis provide comprehensive evidence that CAF+TAU co-supplementation enhances anaerobic performance and reaction time, credibly outperforming CAF or TAU alone. The combination balances CAF’s stimulatory effects with TAU’s neuromodulatory properties, optimizing high-intensity performance while modulating physiological stress responses. However, its effects on aerobic endurance and lactate metabolism remain context-dependent, likely influenced by exercise conditions and individual variability. These findings highlight CAF+TAU as a promising ergogenic aid, particularly for short-duration, power-based activities.

## Supplementary Material

Supplemental Material
